# Reliability and accuracy of the torque applied to osteosynthesis screws by maxillofacial surgeons and residents

**DOI:** 10.1038/s41598-022-18687-7

**Published:** 2022-08-24

**Authors:** Barzi Gareb, Valerie D. M. van Munster, Pieter U. Dijkstra, Ruud R. M. Bos, Arjan Vissink, Nico B. van Bakelen, Baucke van Minnen

**Affiliations:** 1 Department of Oral and Maxillofacial Surgery, University Medical Center Groningen, University of Groningen, Hanzeplein 1, 9713 GZ P.O. Box 30001, 9700 RB, Groningen, The Netherlands; 2 Department of Rehabilitation Medicine, University Medical Center Groningen, University of Groningen, Groningen, The Netherlands

**Keywords:** Preclinical research, Translational research, Implants, Fracture repair, Orthopaedics

## Abstract

Applying the right torque to osteosynthesis screws is important for undisturbed bone healing. This study aimed to compare test–retest and intra-individual reliabilities of the torque applied to 1.5 mm and 2.0 mm osteosynthesis screws by residents and oral and maxillofacial surgeons (OMF-surgeons), to define the reference torque intervals, and to compare reference torque interval compliances. Five experienced OMF-surgeons and 20 residents, 5 of each 4 residency years, were included. Each participant inserted six 1.5 × 4 mm and six 2.0 × 6 mm screws into a preclinical model at two test moments 2 weeks apart (T1 and T2). Participants were blinded for the applied torque. Descriptive statistics, reference intervals, and intra-class correlation coefficients (ICC) were calculated. The OMF-surgeons complied more to the reference intervals (1.5 mm screws: 95% and 2.0 mm screws: 100%) than the residents (82% and 90%, respectively; P = 0.009 and P = 0.007) with the ICCs ranging between 0.85–0.95 and 0.45–0.97, respectively. The residents’ accuracy and reliability were inadequate regarding the 1.5 mm screws but both measures improved at T2 for both screw types compared to T1, indicating a learning effect. Training residents and/or verifying the applied torque by experienced OMF-surgeons remains necessary to achieve high accuracy and reliability, particularly for 1.5 mm screws.

## Introduction

Osteosynthesis screws are the most commonly used implants worldwide^1^. Titanium osteosynthesis systems are important for maxillofacial traumatology, orthognathic surgery and reconstructive surgery^2,3^. The amount of torque applied to the screws contributes to (primary) fracture or osteotomy stability by generating compression and friction between the osteosynthesis system and underlying bone^4,5^. Insufficient screw torque may lead to mobility of bone segments, loosening of screws and disturbed fracture healing, especially on implementing load-bearing osteosyntheses^1^. Applying excessive torque can cause loose screws due to bone stripping or screw breakage^1^.

Currently, applying suitable torque to osteosynthesis screws is based on the surgeon’s “feeling”. Residents are instructed to insert screws with sufficient torque while minimizing the chance of stripping the screw holes or breaking the screws^6^. However, even experienced surgeons are not able to rely fully on their senses^6–9^. A recent systematic review showed that, on average, 26% of all inserted osteosynthesis screws are irreparably damaged or have stripped screw holes, that the awareness of any stripping is poor, and that the variability between surgeons is high^1^. The authors concluded that the optimum torque for different osteosynthesis screws remains unknown and that future research should focus on defining reference torque intervals and developing methods to train clinicians to apply osteosynthesis screws accurately and reliably^1^. Currently, there is no reference torque interval (i.e., a minimum and maximum torque value for safe and adequate bone fixation) for maxillofacial osteosynthesis screws. It is also unknown whether years of experience increases compliance with a predefined reference torque interval (i.e., accuracy) and reliability in the application of osteosynthesis screws.

To enable evidence-based, standardized, and reliable guidance in the application of osteosynthesis screws and to illustrate a simple and low-cost setup to train clinicians, this study aimed to: (1) assess the test–retest and intra-individual reliabilities of the torque applied by residents and experienced OMF-surgeons, (2) define a reference torque interval for the commonly used 1.5 and 2.0 mm osteosynthesis screws and, (3) compare the compliance with the reference torque interval between OMF-surgeons and residents with varying years of experience.

## Materials and methods

**Figure 1:**
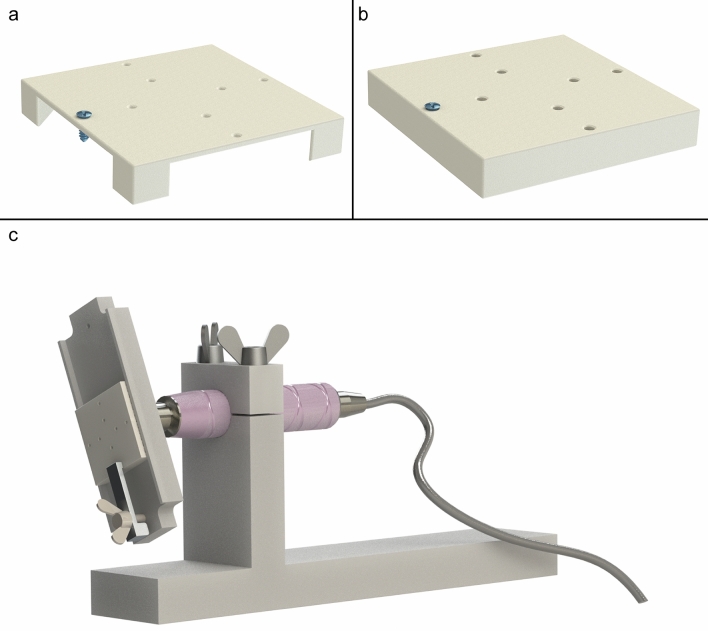
Example of (**a**) a high-pressure laminate (HPL) block with 1 mm thickness used for the 1.5 mm screws and (**b**) an HPL block with 6 mm thickness used for the 2.0 mm screws. Note that the screw goes through the 1 mm thick HPL plate (**a**), i.e. simulating a screw that goes through thin cortical bone (e.g., the anterior wall of the maxillary sinus) while the screw does not go through the 6 mm HPL block (**b**), i.e. simulating a bone screw in cortical bone. (**c**) The test setup with a torque meter with an inserted HPL block. The HPL-block was positioned in such a way that the screw hole of the HPL-block that was used to insert the screw was always aligned with the axis of the torque meter to ensure accurate torque measurement.

The most commonly used titanium osteosynthesis screws in oral and maxillofacial (OMF)-surgery were selected, i.e. the 1.5 × 4 mm and 2.0 × 6 mm KLS Martin MaxDrive® screws (Gebrüder Martin GmbH & Co., Tuttlingen, Germany)^2,3,10^. Predrilled 36 × 36 mm high-pressure laminate (HPL) blocks were chosen as a reproducible model, with a similar elastic modulus as cortical bone^11–13^. Predrilling was performed in a standardized manner with water cooling and using the 1.1 and 1.5 mm diameter drills provided by the manufacturer. To simulate the clinical situation, the thickness of the HPL blocks used for the 1.5 mm screws was 1.0 mm as these screws are commonly used in the midface where the bone is generally thin (e.g., the anterior wall of the maxillary sinus; Fig. 1a). The HPL blocks used for the 2.0 mm screws were 6.0 mm thick as these screws are more commonly used in thick cortical bone (e.g., in the mandible; Fig. 1b)^14^.

**Figure 2:**
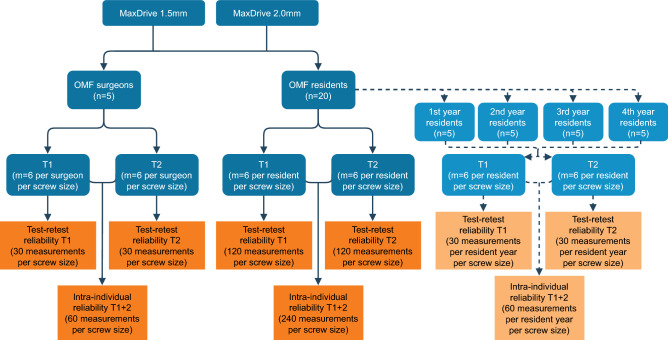
Flowchart of the study procedures to assess the test–retest (at T1 and T2) and intra-individual reliability of the two main groups (i.e., oral and maxillofacial surgeons and residents) and the subgroups (i.e., the different residency years; the dashed lines and lighter colour boxes). *OMF* oral and maxillofacial, *n* number of participants, *m* number of measurements, *T*_*1*_ at baseline, *T*_*2*_ after 2 weeks.

A total of 25 participants were included: five experienced OMF-surgeons (i.e., with many years’ weekly exposure to these osteosynthesis systems in the clinic) and five randomly chosen residents from each of the four residency years (i.e., a total of 20 residents) from University Medical Center Groningen (UMCG, Groningen, the Netherlands) and the Amsterdam University Medical Centers (Amsterdam UMC, the Netherlands), namely Academic Medical Center (AMC) and ‘Vrije Universiteit’ Medical Center (VUmc). The participants were asked to insert 6 screws of each size as they would do in the clinic (‘two-finger tight’) at two test moments (T1 and T2) two weeks apart (Fig. 2). The participants were blinded for the applied torque during both test moments. The burr holes were irrigated with water while inserting the screws to simulate the clinical situation. Saline was avoided to prevent possible corrosion of the test environment. The use of water instead of saline was not expected to influence the test results^10^. The applied torque was measured using a calibrated torque meter (Nemesis Howards Torque Gauge, Smart MT-TH 50 sensor; accuracy 2.5 Nmm; Fig. 1c). Screw breakage and stripped screw holes were recorded.

All the participants were asked for the amount of experience with osteosynthesis systems (also from other disciplines, e.g. orthopaedics, traumatology) and, regarding the residents, the current internship and the number of, and which, internships were completed during their residency.

All methods were carried out in accordance with relevant guidelines and regulations, including the Declaration of Helsinki. The protocol of this study was approved by the Institutional Review Board of the University Medical Center Groningen, the Netherlands. All participants provided written informed consent.

### Sample size calculation

The number of screws of each screw size per participant and per test moment (i.e., m = 6) were derived from the international standard for mechanical testing of bone screws^15^. The number of included participants was based on an a priori performed sample size estimation (1) for group comparisons and (2) to assess intra-individual reliability. The sample size calculation was based on data from a study that assessed differences in the torque applied by 4 OMF-surgeons to 1.5 and 2.0 mm osteosynthesis screws^16^. To provide sufficient power for both the 2.0 and 1.5 mm osteosynthesis screws, the 1.5 mm screw values were used. Using α = 0.05, power = 0.8, effect size = 0.78, and number of groups = 5, resulted in a sample size of 25 participants (i.e., 5 per group)^16^. Regarding the reliability analyses, an expected intra-class correlation coefficient (ICC) of 0.8, and the number of repeated measurements, being 12 per screw size, also resulted in a sample size of 5 participants per group^17^. Therefore, five experienced OMF-surgeons and five randomly chosen residents from each of the four residency years participated, inserting 6 screws of each screw size at two test moments (i.e., a total of 300 measurements per screw size).

### Statistical analyses

All the data were calculated and presented separately for each screw size. The assumptions of normal distribution of continuous data were tested by examining Q–Q plots and histograms, and by performing the Shapiro–Wilk test. Continuous data were presented as mean ± standard deviation (SD) or median (25th to 75th percentile, P_25_–P_75_). Categorical data were reported in numbers and percentages.

Multilevel models were fitted using restricted maximum likelihood estimations that took into account variances between screws within one test moment of a certain participant, between participants within one test moment, and between test moments. A linear multilevel model was fitted for continuous outcome data while a logistic multilevel model was fitted for dichotomous outcome variables. Between-group comparisons (e.g., between OMF-surgeons and residents) were performed using a type III analysis of variance (ANOVA) test.

The test–retest reliability at T_1_, the test–retest reliability at T_2_, and the intra-individual reliability between T_1_ and T_2_ were assessed by calculating the ICC (absolute agreement using a two-way mixed model^17^) with a 95% confidence interval (CI) per group (Fig. 2). An ICC of ≤ 0.50, 0.50–0.75, 0.75–0.90, and ≥ 0.90 was considered as poor, moderate, good or excellent reliability, respectively^18^. A lower limit of the 95% CI of ICC ≥ 0.70 was deemed sufficient for research purposes^18^. The ICC was calculated by dividing the variance components of the participants and the interaction between the participants and the test moments by the total variance^17,19,20^. Bland–Altman plots with limits of agreement were constructed to assess systematic measurement differences^17^.

Due to the lack of a gold standard for osteosynthesis screw torque, the five experienced OMF-surgeons’ measurements (m = 60 screws per screw size) were used to calculate the reference torque intervals for each screw size. We first checked whether the assumption that OMF-surgeons apply osteosynthesis screws consistently was met (i.e., the lower limit of the 95% CI of ICC_intra-individual reliability_ ≥ 0.70). If this assumption was met, the 95% reference intervals of each screw size were calculated based on the experienced OMF-surgeons’ multilevel model data. Here, the variance components of the fixed and random effects were summed (i.e., the total variance), the degrees of freedom were calculated based on the generalized Satterthwaite method (i.e., using the observed variances), and applying the t-values corresponding to the degrees of freedom and α = 0.05, as appropriate^21^. The number and percentage of measurements which complied with the reference intervals were calculated per group and compared between groups.

P ≤ 0.05 (two-tailed) was considered statistically significant. The Bonferroni correction was applied to all the pairwise comparisons to correct for multiple testing. All analyses were performed in R, version 4.0.5, using the *lme4*- and *blandr*-packages^22–24^.

## Results

### Participants’ characteristics

Of the included participants, 16 (64%) were male (all the OMF-surgeons and eleven residents (55%); Table 1). The median age (P_25_–P_75_) was 33.0 years (31.0–38.5; OMF-surgeons: 45.0 years (43.0–63.5); residents: 33.0 years (30.3–34.8)). The OMF-surgeons’ and residents’ experience with osteosynthesis systems was 14.8 (9.5–37.0) and 1.8 (0.2–4.0) years, respectively. Eighty-five per cent of the completed internships had been followed at an academic medical centre.

**Table 1:** Characteristics of the included participants.

	OMF-surgeons (n = 5)	All residents (n = 20)	Residents			
			First year(n = 5)	Second year (n = 5)	Third year (n = 5)	Fourth year (n = 5)
**Gender, n (%)**						
Male	5 (100%)	11 (55%)	4 (80%)	1 (20%)	3 (60%)	3 (60%)
Age, median (P25-P75)	45.0 (43.0–63.5)	33.0 (30.3–34.8)	30.0 (27.0–33.0)	31.0 (27.0–32.0)	36.0 (32.5–38.5)	33.0 (32.0–35.5)
**Medical center, n (%)**						
UMCG	5 (100%)	11 (55%)	3 (60%)	4 (80%)	2 (40%)	2 (40%)
Amsterdam UMC– AMC	0	7 (35%)	2 (40%)	1 (20%)	3 (60%)	1 (20%)
Amsterdam UMC– VUmc	0	2 (10%)	0	0	0	2 (40%)
Experience with osteosynthesis systems in years, median (P_25_–P_75_)	14.8 (9.5–37.0)	1.8 (0.2–4.0)				
**Current internship, n (%)**						
Outpatient clinic	NA	3 (15%)	3 (60%)	0	0	0
Dentoalveolar surgery		1 (5%)	1 (20%)	0	0	0
Trauma surgery		4 (20%)	1 (20%)	1 (20%)	1 (20%)	1 (20%)
Orthognathic surgery		5 (25%)	0	0	3 (60%)	2 (40%)
Implantology		0	0	0	0	0
Oncology		6 (30%)	0	3 (60%)	1 (20%)	2 (40%)
TMJ		1 (5%)	0	1 (20%)	0	0
**Completed internships, n (%)**						
Outpatient clinic	NA	14 (70%)	0	4 (80%)	5 (100%)	5 (100%)
Dentoalveolar surgery		15 (75%)	1 (20%)	4 (80%)	5 (100%)	5 (100%)
Trauma surgery		11 (55%)	0	2 (40%)	4 (80%)	4 (80%)
Orthognathic Surgery		8 (40%)	0	1 (20%)	2 (40%)	3 (60%)
Implantology		9 (45%)	0	0	4 (80%)	5 (100%)
Oncology		7 (35%)	0	0	2 (40%)	3 (60%)
TMJ		4 (20%)	0	1 (20%)	1 (20%)	2 (40%)
Internships followed at academic medical centers, n_academic_/N_total_ (%)*	NA	75/88 (85%)	6/6 (100%)	16/17 (94%)	24/28 (86%)	29/37 (78%)

Bold P-values represent statistically significant differences.

*OMF-surgeons* oral and maxillofacial surgeons, *P*_*25*_*–P75* 25th to 75th percentile, *UMCG* University Medical Center Groningen, *AMC* Academic Medical Center, *VUmc* ‘Vrije Universiteit’ Medical Center, *NA* not applicable, *TMJ* temporomandibular joint.

*Calculated by dividing the number of internships followed at academic medical centres by the total number of internships.

### Torque to osteosynthesis screws

**Figure 3:**
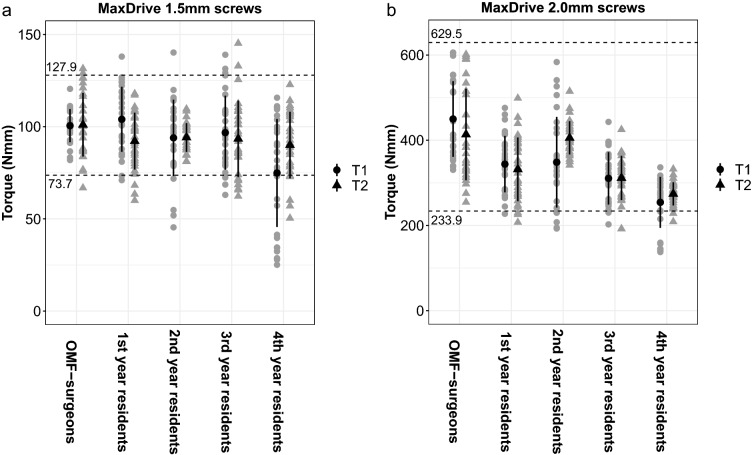
The applied torque to (**a**) 1.5 mm and (**b**) 2.0 mm osteosynthesis screws at T1 and T2. The dotted lines represent the limits of the calculated reference intervals based on the outcomes of the OMF-surgeons accompanied by the corresponding values. Black dots and triangles represent mean values at T1 and T2, respectively, with corresponding standard deviations. *OMF* oral and maxillofacial, *T*_*1*_ at baseline, *T*_*2*_ after 2 weeks.

The OMF-surgeons applied 100.5 ± 9.0 and 101.1 ± 17.2 Nmm torque to the 1.5 mm osteosynthesis screws at T1 and T2, respectively (Table 2, Fig. 3a). The residents applied 92.4 ± 24.6 and 92.4 ± 16.1 Nmm torque to the 1.5 mm osteosynthesis screws at T1 and T2, respectively. The torque applied to the 1.5 mm screws by the residents at both test moments was significantly lower than that applied by the OMF-surgeons. The torque applied by the fourth-year residents at T1 was significantly lower than the first-, second- and third-year residents (Table 2).

**Table 2:** The torque applied by experienced OMF-surgeons and residents at T1 and T2.

	OMF-surgeons	Residents	P-value*	Residents				
				1st year	2nd year	3rd year	4th year	P-value^#^
**MaxDrive 1.5 mm screws, mean** ± **SD (Nmm)**								
T1	100.5 ± 9.0	92.4 ± 24.6	**0.004**	104.0 ± 17.6^c^	93.9 ± 20.7^e^	96.8 ± 20.0f.	74.9 ± 29.3^c,e,f^	** < 0.001**
T2	101.1 ± 17.2	92.4 ± 16.1	**0.011**	92.2 ± 15.4	94.1 ± 7.7	93.4 ± 20.7	90.0 ± 18.0	0.775
**MaxDrive 2.0 mm screws, mean** ± **SD (Nmm)**								
T1	449.8 ± 88.9	314.2 ± 84.0	** < 0.001**	343.9 ± 66.2^c^	348.4 ± 106.4^e^	310.5 ± 61.4f.	254.2 ± 59.9^c,e,f^	** < 0.001**
T2	413.5 ± 107.4	330.7 ± 69.9	** < 0.001**	331.6 ± 74.7^a,c^	405.7 ± 38.9^a,d,e^	311.5 ± 51.8^d,f^	274.1 ± 27.2^c,e,f^	** < 0.001**

The bold P-values represent statistically significant differences.

Each superscript denotes significant differences in the pairwise comparisons (see P-values below): ‘a’ is derived from the pairwise comparison between first- and second-year residents, ‘b’ between first- and third-year residents, ‘c’ between first- and fourth-year residents, ‘d’ between second- and third-year residents, ‘e’ between second- and fourth-year residents, and ‘f’ between third- and fourth-year residents. 1.5 mm screws at T1: ^a^P = 0.502; ^b^P > 0.999; ^**c**^**P < 0.001**; ^d^P > 0.999; ^**e**^**P = 0.008**; ^**f**^**P = 0.001.** 1.5 mm screws at T2: non-significant differences between subgroups and, thus, no pairwise comparisons were performed. 2.0 mm screws at T1: ^a^P > 0.999; ^b^P = 0.551; ^**c**^**P < 0.001**; ^d^P = 0.335; ^**e**^**P < 0.001**; ^**f**^**P = 0.029.** 2.0 mm screws at T2: ^**a**^**P < 0.001**; ^b^P = 0.790; ^**c**^**P < 0.001**; ^**d**^**P < 0.001**; ^**e**^**P < 0.001**; ^**f**^**P = 0.034.**

*SD* standard deviation; *OMF-surgeons* oral and maxillofacial surgeons.

*Comparison between OMF-surgeons and residents.

^#^Comparison between the residency years.

The OMF-surgeons applied 449.8 ± 88.9 and 413.5 ± 107.4 Nmm torque to the 2.0 mm osteosynthesis screws at T1 and T2, respectively (Table 2, Fig. 3b). The residents applied 314.2 ± 84.0 and 330.7 ± 69.9 Nmm torque to the 2.0 mm osteosynthesis screws at T1 and T2, respectively. The torque applied to the 2.0 mm screws by the residents at both test moments was significantly lower than the torque applied by the OMF-surgeons. The torque applied by the fourth-year residents at T1 and T2 was significantly lower than the first-, second- and third-year residents.

### Test–retest and intra-individual reliability

**Figure 4:**
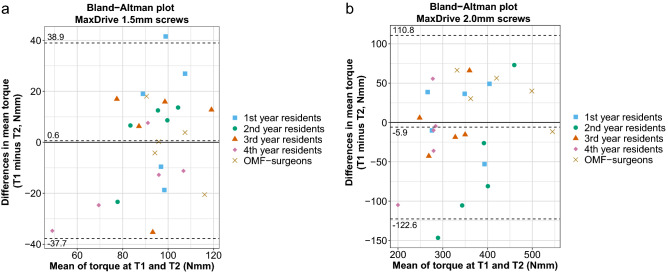
Bland–Altman plots of the (**a**) 1.5 mm and (**b**) 2.0 mm osteosynthesis screws. The dotted lines represent the lower and upper limits of agreement and the systematic difference accompanied by the corresponding values. The 95% CI of the systematic difference of the 1.5 mm screws is − 7.4 to 8.7 Nmm, and that of the 2.0 mm screws is − 30.5 to 18.7 Nmm. *OMF *oral and maxillofacial, *CI* confidence interval.

The OMF-surgeons achieved moderate to good test–retest and intra-individual reliability for the 1.5 mm screws (Table 3). The residents (i.e., as one group) achieved good to excellent test–retest and intra-individual reliability for the 1.5 mm screws. The subgroup analysis showed that the test–retest and the intra-individual reliability of the first- and second-year residents ranged from poor to moderate. In contrast, the third- and fourth-year residents achieved moderate to good reliabilities (Table 3). The Bland–Altman plot (Fig. 4a) demonstrated a systematic difference of 0.6 Nmm (limits of agreement (LOA) 38.9 to − 37.7 Nmm).

**Table 3:** Test–retest reliability (at T1 and T2) and intra-individual reliability between T1 and T2.

	OMF-surgeons	Residents	Residents			
			1st year	2nd year	3rd year	4th year
**MaxDrive 1.5 mm screws, ICC (95% CI)**						
Test–retest reliability T1	0.85 (0.53;0.99)	**0.95 (0.91;0.98)**	0.89 (0.65;0.99)	0.83 (0.36;0.98)	**0.96 (0.86;0.99)**	**0.97 (0.90;0.99)**
Test–retest reliability T2	**0.95 (0.83;0.99)**	**0.91 (0.84;0.96)**	0.90 (0.67;0.99)	0.90 (0.68;0.99)	**0.92 (0.71;0.99)**	**0.96 (0.88;0.99)**
Intra-individual reliability (T1–T2)	**0.93 (0.77;0.99)**	**0.92 (0.85;0.96)**	0.45 (0.00;0.93)	0.87 (0.61;0.99)	**0.92 (0.75;0.99)**	**0.97 (0.90;0.99)**
**MaxDrive 2.0 mm screws, ICC (95% CI)**						
Test–retest reliability T1	**0.92 (0.73;0.99)**	**0.96 (0.92;0.98)**	**0.94 (0.98;0.99)**	0.83 (0.44;0.98)	0.89 (0.62;0.99)	0.86 (0.54;0.98)
Test–retest reliability T2	**0.94 (0.81;0.99)**	**0.97 (0.94;0.99)**	**0.96 (0.87;0.99)**	**0.96 (0.87;0.99)**	**0.97 (0.90;0.99)**	**0.98 (0.93;0.99)**
Intra-individual reliability (T1–T2)	**0.96 (0.89;0.99)**	**0.96 (0.93;0.98)**	**0.97 (0.90;0.99)**	**0.92 (0.76;0.99)**	**0.96 (0.87;0.99)**	**0.92 (0.75;0.99)**

The bold values indicate sufficient reliability (i.e., ICC ≥ 0.7).

*ICC* intra-class correlation coefficient, *95% CI* 95% confidence interval, *OMF-surgeons* oral and maxillofacial surgeons.

The OMF-surgeons achieved moderate to good test–retest and intra-individual reliability for the 2.0 mm screws. The residents achieved excellent test–retest and intra-individual reliability for the 2.0 mm screws. The subgroup analysis showed that the T_1_ test–retest reliability of the second-, third-, and fourth-year residents ranged from poor to moderate. However, the T_2_ test–retest reliability and intra-individual reliability of these subgroups increased to good–excellent reliability (Table 3). The Bland–Altman plot (Fig. 4b) showed a systematic difference of -5.9 Nmm (LOA 110.8 to − 122.7Nmm).

### Reference intervals and complications

Since the assumptions that OMF-surgeons apply osteosynthesis screws consistently were met for both the 1.5 and 2.0 mm osteosynthesis screws, reference intervals for both screw sizes could be calculated ranging from 73.7 to 127.9 Nmm for the 1.5 mm screws (Fig. 3a) and from 233.9 to 629.5 Nmm for the 2.0 mm screws (Fig. 3b).

The OMF-surgeons’ compliance with the reference torque interval for the 1.5 mm screws was 57 (95%) whereas the residents’ compliance was 195 (82%) (P = 0.009; Table 4; Fig. 3a). The first- and second-year residents complied with the reference interval significantly more often than the third- and fourth-year residents (Table 4). The compliance to the reference interval increased from 82% at T1 to 86% at T2. Screw hole stripping with the 1.5 mm screws was similar among the OMF-surgeons and residents (Table 4). The second-year residents had the highest proportion of stripped screw holes (17%).

**Table 4:** The compliance with the reference intervals and the number of complications during osteosynthesis screw insertion.

	OMF-surgeons	Residents	P-value*	Residents				
				1st year	2nd year	3rd year	4th year	P-value^#^
**MaxDrive 1.5 mm screws, n (%)**								
Reference interval compliance^†^	57 (95%)	195 (82%)	**0.009**	54 (90%)	55 (92%)^d,e^	43 (72%)^d^	43 (72%)^e^	**0.002**
Stripped screw holes	1 (2%)	14 (6%)	0.319	0 (0%)^a^	10 (17%)^a,e^	4 (7%)	0 (0%)^e^	** < 0.001**
Broken screws	0 (0%)	0 (0%)	NA	0 (0%)	0 (0%)	0 (0%)	0 (0%)	NA
**MaxDrive 2.0 mm screws, n (%)**								
Reference interval compliance^§^	60 (100%)	215 (90%)	**0.007**	56 (93%)	54 (90%)	56 (93%)	49 (82%)	0.168
Stripped screw holes	1 (2%)	0 (0%)	0.200	0 (0%)	0 (0%)	0 (0%)	0 (0%)	NA
Broken screws	0 (0%)	0 (0%)	NA	0 (0%)	0 (0%)	0 (0%)	0 (0%)	NA

The bold P-values represent statistically significant differences.

Each superscript denotes significant differences in the pairwise comparisons (see P-values below): ‘a’ is derived from the pairwise comparison between first- and second-year residents, ‘b’ between first- and third-year residents, ‘c’ between first- and fourth-year residents, ‘d’ between second- and third-year residents, ‘e’ between second- and fourth-year residents, and ‘f’ between third- and fourth-year residents. 1.5 mm screw reference interval compliance: ^a^P > 0.999; ^b^P = 0.064; ^c^P = 0.064; ^**d**^**P = 0.028**; ^**e**^**P = 0.028**; ^f^P > 0.999; stripped screw holes, ^**a**^**P = 0.008;** ^b^P = 0.712; ^c^P = NA; ^d^P = 0.920; ^**e**^**P = 0.008;** ^f^P = 0.712; broken screws: NA. 2.0 mm screw reference interval compliance: non-significant differences between subgroups and, thus, no pairwise comparisons were performed; stripped screw holes: NA; broken screws: NA.

*Comparison between OMF-surgeons and residents.

^#^Comparison between the residency years.

^†^Reference interval 1.5 mm screws: 73.7–127.9 Nmm.

^§^Reference interval 2.0 mm screws: 233.9–629.5 Nmm.

*OMF-surgeons* oral and maxillofacial surgeons, *NA* not applicable.

The OMF-surgeons complied with the reference torque interval on applying all the 2.0 mm screws (Table 4; Fig. 3b). The residents’ compliance with the 2.0 mm screw reference interval was 215 (90%) (P = 0.007; Table 4; Fig. 3b). Compliance with the 2.0 mm screw reference interval was similar among all the residents (Table 4). The reference interval compliance increased from 88% at T1 to 95% at T2. The OMF-surgeons and residents’ screw hole stripping with the 2.0 mm screws was similar.

## Discussion

This study shows a clear effect of “learning-by-doing”, with increased compliance to the reference torque intervals and reliability for both 1.5 and 2.0 mm osteosynthesis screws at T2 compared to T1. The senior residents showed higher reliability but lower compliance with the reference torque interval compared to the junior residents. Thus, despite the residency year, it is still necessary to train residents and/or to verify the applied torque by experienced OMF-surgeons remains necessary to utilize the full potential osteosynthesis systems.

A simulated learning environment is very suitable for acquiring the “feeling” of adequate screw fixation with sufficient tightness and when a screw hole will strip. This study shows that learning-by-doing increases both the test–retest reliability and compliance with the reference torque intervals for both 1.5 and 2.0 mm screws. Although first- and second-year residents showed an increase in reliability with the 1.5 mm screws at T2 compared to T1, these reliabilities were still insufficient at T2 (i.e. ICC < 0.7). All the other groups with insufficient applied torque reliability at T1 increased their reliability to a sufficient level at T2. These results indicate that this test setup has a learning effect on OMF clinicians resulting in increased reliability and accuracy for both screw types. Since bone stripping and screw breakage are more likely to occur when the difference between the torque applied to the screws for adequate fixation (i.e., hand-tight) and the maximum allowed torque (i.e., torque up to screw breakage) is small^25^ as well as that this setup can increase both accuracy and reliability of the applied torque, this setup is appropriate for educational purposes.

At first glance, the calculated reference intervals for both screw sizes may seem wide. The reference intervals are wide because the dispersion around the mean torque applied by the maxillofacial surgeons (i.e., the standard deviation and, thus, the variance) is relatively large. The high variability of torque applied to osteosynthesis screws between surgeons has also been reported in literature previously^1^. However, as each surgeon applied the torque consistently (i.e., the intra-individual reliability was good to excellent) and there were no signs of systematic difference between T1 and T2 in the Bland–Altman plots, the measured variability between surgeons is, thus, part of the actual application of screws. Due to the higher maximum torque needed to adequately insert the 2.0 mm screws, which in turn inevitably results in a loss in precision^16^, the reference interval of the 2.0 mm screws is much wider than that of the 1.5 mm screws.

The reliability and compliance with the 2.0 mm screw reference torque interval were generally better than the 1.5 mm screws as the latter are more prone to errors (i.e., too little or much applied torque). An explanation for these differences is that the tactile feedback is higher when applying 2.0 mm screws^1,16^, as shown by other studies that increasing tactile or visual feedback results in increased accuracy and the ability to predict screw hole stripping^1^. Therefore, complying with the 1.5 mm screw reference interval requires a higher degree of accuracy. Thus, although training is beneficial for both screw sizes, training of the applied torque to 1.5 mm screws is, in particular, needed.

Our study shows that this combination of compliance with the reference interval and residents’ intra-individual reliability is currently inadequate for 1.5 mm screws. Although the first- and second-year residents showed higher compliance with the reference interval, the intra-individual reliability of both subgroups was poor and moderate, respectively. The third- and fourth-year residents demonstrated good intra-individual reliability but poorer compliance with the reference interval. On the other hand, regarding the 2.0 mm osteosynthesis screws, the first-, second- and third-year residents had good intra-individual reliability and high compliance with the reference interval. The fourth-year residents displayed good intra-individual reliability but applied too little torque to a substantial proportion of the screws. A post hoc analysis of the fourth-year residents’ insertions showed that the torque of 10/17 (59%) of the 1.5 mm and 7/11 (64%) of the 2.0 mm screws was insufficient. A recent review also showed substantial between-surgeon variability in the application of osteosynthesis screws^1^. Therefore, regardless of the residency year, training residents (e.g., by using this test setup) and/or verification of the applied torque by experienced OMF-surgeons remains necessary when applying osteosynthesis systems.

Stripping of the screw holes only occurred on inserting the 1.5 mm screws. Interestingly, the second-year residents showed the highest proportion of stripped screw holes but with the highest compliance with the 1.5 mm reference interval. An explanation is that this was caused by the self-tapping technique for osteosynthesis screws, i.e. tightening the screws by clockwise rotation, followed by loosening the screws a bit by rotating anti-clockwise and then tightening the screws further. This technique is necessary to lower the torsional resistance when applying osteosynthesis screws as well as to remove debris that is formed on self-tapping the screw holes. However, when this technique is executed too forcefully, the screw holes get stripped without having applied excess torque^1^. All the (sub)groups’ stripping rates remained lower compared to the average stripping rate (26%) reported in the literature^1^, probably because the screws used in this study were smaller, necessitating less torque.

The calculated reference intervals and the reported learning effect indicate that training clinicians (e.g., during the residency period, seminars or courses) with this simple, yet effective test setup has the potential to improve the effectivity of osteosynthesis systems. This has the potential to enhance patient care quality by increasing fracture or osteotomy stability, resulting in less compromised healing, and reducing the need for emergency screws following the stripping of bone intraoperatively, with a corresponding reduction in operation time and costs.

The osteosynthesis screws included in this study are used for fixating fractures and osteotomies in different locations of the facial skeleton, e.g., the crista zygomaticoalveolaris, anterior wall of the maxillary sinus, and mandible. These maxillofacial bones have different mechanical properties^12,13,26,27^. The HPL-blocks used in this study have mechanical properties within the known mechanical property range of maxillary and mandibular bones^11^, making them a suitable bone simulation model. However, the translation of the reference intervals to the clinical setting remains uncertain due to in vivo variabilities in bone density and thickness. Therefore, we advocate that translation of the reference intervals to a clinical setting should not be done until in vivo validation of the calculated reference intervals has been performed.

Although this study focused on maxillofacial osteosynthesis systems, the results of this study also seem applicable to other disciplines that use osteosynthesis systems, e.g., orthopaedic and trauma surgery. A recent systematic review showed that, on average, 26% of all inserted osteosynthesis screws by experienced orthopaedic and trauma surgeons are irreparably damaged or have stripped screw holes^1^. Currently, it remains unknown how residents of these disciplines perform. The authors of that review indicated that there is a need for defining reference torque intervals and that future research should focus on developing methods to train clinicians to apply osteosynthesis screws accurately and reliably^1^. The test setup presented in this study can be easily adjusted by using a different torque meter (i.e., that can measure higher torque for larger screws) and different HPL-blocks, making this test setup useful for educational purposes with different sizes of osteosynthesis systems.

The strengths of this study are the simple, effective and standardized test setup, blinding all the participants to the applied torque, and the thorough study design (i.e., test–retest reliability at T_1_ and T_2_, and intra-individual reliability between T_1_ and T_2_). The presented low-cost test setup can be easily fabricated for educational purposes. Furthermore, commonly used osteosynthesis screws were applied to a standardized bone model. A limitation of this study is that, although we used a suitable bone simulation model, translation of the reference intervals to the clinical setting remains uncertain due to in vivo variabilities in bone density and thickness. Moreover, bone blocks were not used because the variability in bone mineral density, cortical and spongious bone layer thickness, and block dimensions impede their use as a standardized and reproducible model since reliability assessment is then uncertain. Another limitation is the lack of a gold standard for torques applied to screws. We, therefore, determined reference intervals based on the torque values of experienced OMF-surgeons. The participating surgeons have many years of experience with osteosynthesis systems in the clinic. However, another group of OMF-surgeons might have given other reference intervals. External validation of the defined reference intervals by future research is therefore desired. Finally, since the error of the torque meter is a fixed absolute value (i.e., 2.5 Nmm), the relative error increases as the measured torque decreases. However, this study aimed to assess and compare the reliability and accuracy of maxillofacial surgeons and residents as well as to provide a simple and low-cost, yet effective, setup that can be used to train residents to increase the reliability and accuracy of the torque applied to osteosynthesis screws. The results show that the used torque meter can measure with sufficient accuracy and precision to assess the reliability, accuracy and learning-effect over time, and, thus, suits the aims of this study.

## Conclusions

This study shows a learning effect on using a simple and low-cost, yet effective, setup resulting in increased compliance with the reference torque intervals and reliability regarding both 1.5 mm and 2.0 mm osteosynthesis screws. Senior residents showed higher reliability but lower compliance with the reference torque intervals compared to junior residents. The combination of high accuracy and reliability by residents was insufficient for 1.5 mm screws. Thus, despite the residency year, training and/or verification of the applied torque by experienced OMF-surgeons is still necessary for residents to utilize osteosynthesis systems to their fullest potential.

## Data Availability

The datasets used and/or analysed during the current study available from the corresponding author on reasonable request.
